# Transmission dynamics of foot and mouth disease in selected outbreak areas of northwest Ethiopia

**DOI:** 10.1017/S0950268819000803

**Published:** 2019-05-09

**Authors:** B. Tadesse, W. Molla, A. Mengsitu, W. T. Jemberu

**Affiliations:** 1 University of Gondar, College of Veterinary Medicine and Animal Sciences, Department of Veterinary Epidemiology and Public Health, P.O. Box. 196, Gondar, Ethiopia; 2 Amedguya Sheep Breed Improvement and Multiplication Center, P.O. Box. 30, North Shoa, Ethiopia

**Keywords:** Cattle, foot and mouth disease, northwest Ethiopia, reproduction ratio, transmission rate

## Abstract

Foot and mouth disease (FMD) is a highly contagious and economically important disease of cloven-hoofed animals, which is endemic in Ethiopia. An outbreak follow-up study was undertaken to quantify the transmission parameters of FMD in the crop–livestock mixed (CLM) system and commercial dairy farms in selected areas of northwest Ethiopia. The transmission parameters were quantified using a generalised linear model (GLM) based on a susceptible–infectious–recovered (SIR) epidemic model. The per day average transmission rate between animals was 0.26 (95% CI 0.22–0.32) and 0.33 (95% CI 0.21–0.57) in the CLM system and in the commercial dairy farms, respectively. The average basic reproduction ratio of FMD was 1.68 (95% CI 1.42–2.07) in the CLM system and 1.98 (95% CI 1.26–3.42) in the commercial dairy farms. The medium per day transmission rate and moderate basic reproduction ratio observed in this study indicated that a vaccination coverage needed to stop transmission of the disease in these populations might not be very high.

## Introduction

Ethiopia has approximately 59.5 million cattle, 30.7 million sheep and 30.2 million goats [1]. Livestock production in Ethiopia broadly classified into three systems: crop–livestock mixed (CLM), pastoral and market-oriented production systems. The dominant production system is the CLM system, which accounts for about 80–85% of the cattle population [2]. The pastoral production system is the second most dominant farming system, which is commonly practiced in the arid and semiarid peripheral parts of the country, and accounts for about 15–20% of the cattle population [2]. The third type of production system is market-oriented production in urban and peri-urban parts of the country, which is very small and primarily consists of dairy cattle and to some extent feedlots. The contribution of the livestock sector to the national economy is minimal compared to its potential. One of the main reasons for this is the widespread occurrence of many infectious diseases, such as foot and mouth disease (FMD), which drastically reduces the production and productivity of livestock [3].

FMD is a contagious trans-boundary and economically devastating viral disease of cloven-hoofed animals including both domestics and wildlife species [4, 5]. Foot and mouth disease virus (FMDV) that is classified within the genus *Aphtovirus* and family *Picornaviridae* causes the disease. FMDV consists of seven different serotypes (A, O, C, Asia1, SAT (South African territories) 1, SAT2 and SAT3) with many subtypes [6]. It is characterised by vesicular eruptions in the oral cavity, foot and udder; these lesions are associated with fever, lameness, salivation and anorexia [7]. The virus can be transmitted either directly, e.g. via contact with an infected host/s [8, 9], or indirectly, e.g. via contact with a contaminated environment with FMDV-infected secretions and excretions [10, 11].

The transmission dynamics of infectious diseases like FMD have important effects on the epidemiology of the disease and measures that can be taken to control them. A parameter often used to describe the magnitude of transmission is the basic reproduction ratio (*R*_0_). The *R*_0_ is defined as the average number of secondary infections caused by one typical infectious individual in a fully susceptible population during its entire infectious period [12]. Whether an outbreak spreads or dies out depends on whether the *R*_0_ is greater than or less than one. If *R*_0_ exceeds one, an infected animal infects on average more than one susceptible animal, and thus it may cause a major outbreak, but if R_0_ is smaller than one, the disease will die before being generalised to a major outbreak [13, 14]. A limitation of *R*_0_, however, is that it does not include a time factor, which is important in epidemic modelling to analyse the course of the epidemic. A suitable parameter to use in modelling that does have a time dimension is the transmission rate (*β*), which is defined by the average number of new infections caused by one infectious individual per unit of time [15].

To understand the transmission behaviour of FMDV and be able to predict its transmission dynamics, quantification of FMDV transmission parameter is essential. Quantification of *R*_0_ for FMDV can be performed by using field data [16] and data from animal experiments [17]. Different authors determine *R*_0_ for FMD in different settings using different approaches. For example, *R*_0_ was quantified from the final size of infection in sheep as 1.1 [9], from transmission experiment in cattle as 2.52 [8] in the Netherlands and from sero-prevalence data in cattle in Ethiopia as 1.45 [18].

FMDV is endemic in Ethiopia in all production systems since it was first recorded in 1957 [19] and a large number of outbreaks were reported every year [20, 21]. Based on data over the years 2007–2012, the annual district-level incidence of FMD outbreaks was estimated at 0.24, 0.39 and 0.85 per district year in the CLM, pastoral and market-oriented systems, that are caused by serotypes O, A, SAT 2 and SAT 1 [22]. Different studies undertaken on FMD so far also revealed the existence of the disease in different parts of the country, with different sero-prevalence ranging from 5.6% to 24.2% [21, 23, 24, 25, 26, 27, 28]. Quantitative information on the transmission dynamics of FMD is essential in order to make sound decisions about its control. Despite a large number of FMD outbreaks reported in Ethiopia every year, its transmission dynamics have not been properly quantified before. Therefore, the current study was undertaken with the objective of determining the transmission rate and reproduction ratio of FMD using field outbreak data collected from some selected areas of northwest Ethiopia.

## Materials and methods

### Description of the study area

**Fig. 1.**
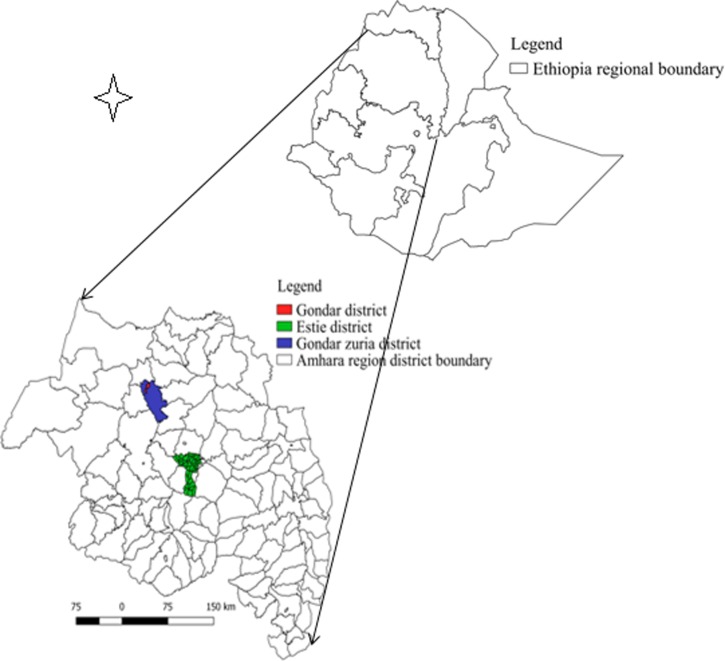
Map of Amhara region showing FMD transmission study sites.

The study was conducted in two districts in the CLM production system (Estie district of South Gondar zone and Gondar zuria district of North Gondar zone) and five commercial dairy farms in Gondar town (Fig. 1). These districts and Gondar town were selected for study because of the current FMD outbreak during the time.

### Study herds, animals and animals contact patterns

The study populations were household herds (group of animals that may comprise cattle, sheep or goat and owned by a household for subsistence) in FMD outbreak-affected kebeles (the smallest administrative unit in Ethiopia) for CLM system and FMD-affected dairy herds in the commercial dairy system. A total of 1296 herds: 745 herds from Estie district, 546 herds from Gondar zuria district and five commercial dairy farms from Gondar town were used for the transmission study. These herds comprise 16 984 animals: 8706 animals from Estie district, 8190 animals from Gondar zuria district and 88 animals from Gondar town commercial dairy farms.

The animals' contact network depends on a number of factors, including housing system, herd size, nature of grazing lands, watering points, herd density and availability of main livestock transporting roads within the district. Animals in Estie and Gondar zuria districts were managed extensively, whereas the five dairy farms in Gondar town managed intensively. In Estie district, animals from different kebeles regularly mix at communal grazing areas and watering points, so that they all were considered as one epidemiological unit for studying the transmission of FMD. While the two kebeles (Tsion and Enfranz) included in the study from Gondar zuria district are separated by a mountain range that limits animal contact between these two kebeles, so they were taken as two different epidemiological units. Each commercial dairy farm was taken as a separate epidemiological unit. Most of the animals in the CLM type of herds are local Zebu breed, while animals in the commercial herds are Holstein-Friesian local Zebu cross.

### Outbreak follow-up and infection status of animals

Monitoring of the FMD outbreaks was done from September 2017 to May 2018 in Estie district (in three affected kebeles), Gondar zuria district (in two affected kebeles) and Gondar town (in five affected dairy farms) to study the transmission dynamics of FMD outbreaks. Follow-up of herds and field case observations were conducted in the outbreak-affected kebeles during the active outbreak periods.

FMD outbreak-affected kebeles in each district were identified from the records of district veterinary offices. In each district, the follow-up study started immediately after the FMD index case observed in the districts. In kebeles affected by the outbreak, herds were visited once per week to check the occurrence of FMD cases until the end of the outbreak. If a case was found in a herd, the infection chain within the herd was monitored by visiting the affected herd twice a week until the end of the outbreak and the FMD status (susceptible, infected and recovered) of all animals were recorded. Animals showing lameness, salivation (drooling), smacking of the lips, grinding of teeth, vesicles/lesions in the mouth (on the tongue, gum, cheeks, lips), unwillingness to move or stand, significant drop in milk production, and high morbidity and low mortality in the herd were considered FMD cases [29]. Herd owners were asked to record new cases each day and report to the investigator. At the start of the study, all cattle, sheep and goat in the affected kebele in the CLM production system were assumed susceptible. In the commercial dairy farms, all animals on the farm were taken as susceptible. In this study, animals were considered infected when they showed clinical signs of FMD during the follow-up period of the outbreak. Animals were registered as a new case on the date they were reported or seen with FMD clinical signs and as infectious on the same day [30]. Infected cattle were assumed to stay infectious on average for 6 days, whereas sheep and goats were assumed to stay infectious on average for 28 days. These infectious periods were determined by taking into account the duration of virus isolation in blood and oropharyngeal swab for 5.5 (95% CI 4.5−6.7) days [11] and in saliva for 10 days [31] for cattle, and 52 days [32] and 28 days (95% CI 19–42) [17] for sheep. Animals that died before the completion of the infectious period were considered infectious only for the days they lived after being infectious.

Parallel to the transmission follow-up, samples from FMD clinically infected animals (vesicular fluids and epithelium from ruptured vesicles in the oral cavity and inter-digital space) were collected and tested using antigen detection ELISA following the procedure described by OIE [33] at the National Animal Health Diagnostic and Investigation Center, Sebeta, Ethiopia to confirm that the clinically observed disease was truly FMD.

### Quantification of FMD transmission parameters

The transmission parameters were estimated based on a SIR epidemic model in which individuals in the population were categorised either susceptible (S), infectious (I) or recovered and immune (R). During the study, the numbers of infectious, susceptible and recovered animals observed in each herd were recorded at the start of each observation interval. Transmission of FMDV between animals was estimated from the relationship between the number of infectious animals at the start of the time interval and the number of newly infected animals at the end of the time interval.

The transmission rate parameter was estimated by a GLM [11, 34, 35] based on a stochastic SIR epidemic model [36] in which transmission of FMD between individuals are described by the change in the number of susceptible, infectious and recovered animals. In the SIR model, susceptible animal becomes infected with a rate of
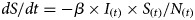


in which *β* is the transmission rate parameter, *S*_*t*_ is the number of susceptible animals at time *t*, *I*_*t*_ is the number of infectious animals at time *t* and *N*_*t*_ is the total number of animals at time *t*. The number of infectious contacts encountered by one individual in a period of length Δ*t* follows a Poisson distribution with a parameter (*β*×(*I*_*t*_) × Δ*t*/*N*_*t*_). In the described model, the probability for a susceptible animal to escape infection during a period Δ*t* is given by *e*^−*β*×*I*×Δ*t*/*N*^ and the probability to become infected is therefore 1−*e*^−*β*×*I*×Δ*t*/*N*^. This implies that the number of new cases in a period Δ*t* follows a binomial distribution with binomial total *S* and the above expressed probability. The expected number of new cases (*C*) in a unit of time is then *E*(*C*) = *S* (1−e^−*β*×*I*×Δ*t*/*N*^) [36, 37]. The between-animals transmission rate *β* (*β* = *e^b^*, where *b* is the coefficient of the intercept of the model) was quantified using GLM [38] with number of new cases as dependent variable, complementary log log-LINK function, *S* as binomial total and the natural logarithm of (*I*×Δ*t*/*N*) as the offset variable using the GLM expression cloglog E(C_*t*_/S_*t*_) = ln(*β*) + ln(*I*×Δ*t/N*). Finally, *R*_0_ was estimated by multiplying *β* with the average length of the infectious period [11, 36].

### Data management and statistical analysis

The data collected from the field case follow-up was entered into an Excel spreadsheet and the data were checked for errors of entry and then imported to STATA version 12 for analysis. The GLM was used to estimate the transmission rates. Statistical analyses were also conducted to test the significance of differences in transmission rate between production systems. A *P*-value <0.05 was considered as significant in the comparisons.

## Results

### Herd structure and FMD occurrence

For studying the transmission dynamics of FMD, a total of 16 984 individual animals (13 935 heads of cattle and 3049 heads of sheep and goats) from 1296 herds were monitored for FMD occurrence. Among all these animals monitored, 16 896 (13 847 cattle and 3049 sheep and goats) of them were kept in 1291 herds in the CLM production system and the remaining 88 animals were kept in five herds in the commercial dairy production system.

**Fig. 2.**
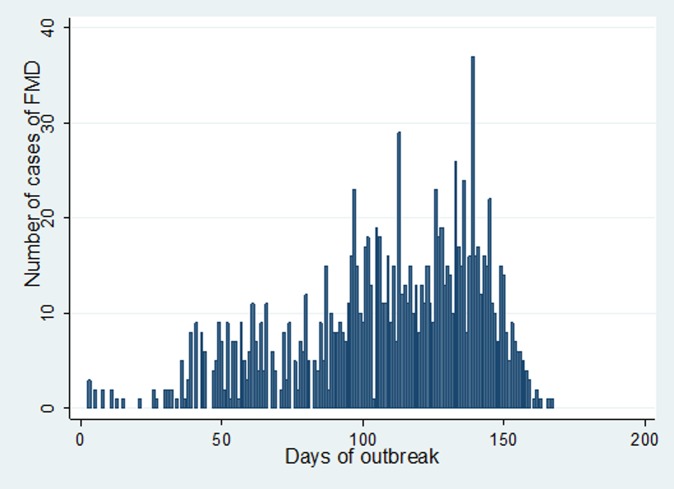
Epidemic curve of FMD in Estie district, northwest Ethiopia in 2017/18.

**Fig. 3.**
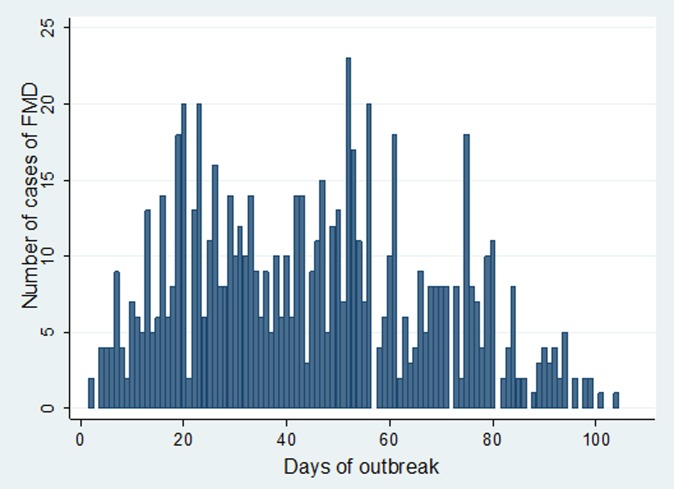
Epidemic curve of FMD in Enfranz area, northwest Ethiopia in 2017/18.

**Fig. 4.**
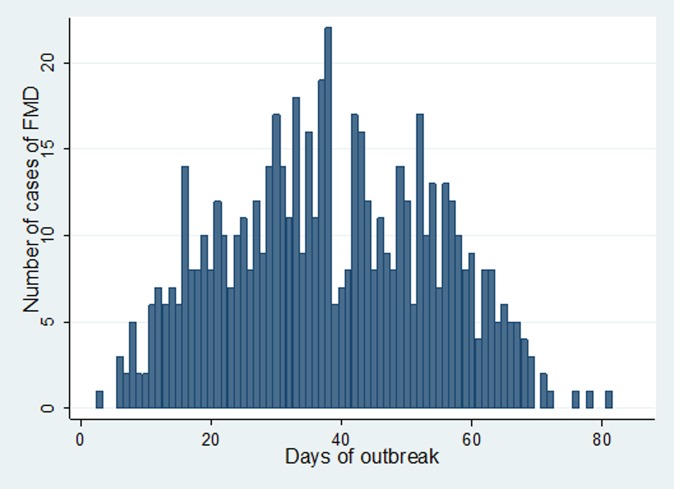
Epidemic curve of FMD in Tsion area, northwest Ethiopia in 2017/18.

In Estie district, the FMD outbreak started at the end of August 2017 and continued until the end of February 2018, but in Gondar zuria district and Gondar town, the outbreak started at the first week of October 2017 and ended in the last week of February 2018. The epidemic curve of FMD outbreaks in Estie district and in Enfranz and Tsion Kebeles of Gondar zuria district are presented in Figs 2, 3 and 4, respectively. The outbreaks in all study areas were confirmed as FMD and the causal virus was identified as serotype O by antigen detection ELISA.

### Transmission parameters of FMD

The transmission rate between animals in the CLM production system was 0.31 (95% CI 0.27–0.36), 0.23 (95% CI 0.19–0.31) and 0.24 (95% CI 0.20–0.31) per day in Estie, Tsion and Enfranz, respectively, whereas in the commercial dairy farms, it varied from 0.21 (95% CI 0.09–0.48) to 0.42 (95% CI 0.23–0.73) in the different farms (Table 1). The average transmission rate between animals in the commercial dairy farms was 0.33 (0.26–0.42) per day, but it was 0.26 (95% CI 0.22–0.32) in the CLM production system (Table 1). There was a significant difference (*P* < 0.05) in the average transmission rate between animals in the CLM production system and commercial dairy farms. A reproduction ratio of 2.12, 1.45 and 1.52 between animals was estimated in the CLM production system in Estie, Tsion and Enfranz areas, respectively, while the average *R*_0_ value for CLM production system was 1.68. In commercial dairy farms, *R*_0_ values between animals were varying from 1.26 to 2.52, while the average *R*_0_ value was 1.98 (Table 1).

**Table 1.** Transmission rate parameter and reproduction ratio of FMDV in CLM herds and within the five commercial dairy farms in northwest Ethiopia during the 2017/18 outbreaks

Production system	District/farm	No. of cases	*β* (95% CI) per day	Average infectious period in days	*R*_0_ (95% CI)
CLM	Estie	1697	0.31 (0.27–0.36)	6.84	2.12 (1.85–2.46)
	Tsion	775	0.23 (0.19–0.27)	6.20	1.45 (1.2–1.67)
	Enfranz	1003	0.24 (0.21–0.27)	6.34	1.52 (1.33–1.71)
	**Average of all districts**	**3475**	**0.26 (0.22–0.32)**	**6.46**	**1.68 (1.42–2.07)**
Commercial dairy farms	Farm 1	7	0.32 (0.2–0.57)	6	1.92 (1.2–3.42)
	Farm 2	8	0.42 (0.23–0.73)	6	2.52 (1.38–4.38)
	Farm 3	8	0.21 (0.09–0.48)	6	1.26 (0.54–2.88)
	Farm 4	11	0.36 (0.24–0.6)	6	2.16 (1.44–3.6)
	Farm 5	14	0.34 (0.24–0.51)	6	2.04 (1.44–3.06)
	**Average of all farms**	**48**	**0.33 (0.21–0.57)**	**6**	**1.98 (1.26–3.42)**

*P* = 0.001 for difference in average transmission rate between animals in the CLM production system and commercial dairy farms.

## Discussion

To the best of our knowledge, this is the first study in Ethiopia in which the transmission parameters for FMD have been quantified from real outbreak situations in the field. This knowledge is helpful to understand the disease transmission dynamics and design sets of measures that efficiently control the disease.

In this study, the average between animal transmission of FMDV was estimated at 0.26/day for CLM system and 0.33/day for the commercial farms with some variations among the epidemiological units within each system (0.23–0.31/day in the CLM and 0.21–0.42/ day in commercial dairy farms). The average per day transmission rates of FMD between animals estimated in this study were higher than the transmission rate from sheep to cattle (0.037) reported previously [17]. This might be because of the difference in the study population and management of animals.

It is widely believed that FMDV is transmitted from infected to susceptible hosts directly or indirectly through contaminated environment and/or fomites [8, 11]. However, in this study, it was impossible to identify clearly, which specific infected animal transmitted the infection to which susceptible animal in the transmission chain due to the mixing up of animals at common grazing areas and watering points and hence it was difficult to separate the transmission as direct and or indirect (environmental). Due to the mixed nature of herds (i.e. cattle, sheep and goats) in the CLM system, it was also not possible to know from which species an animal acquired the infection and the estimated transmission rate includes both the intraspecies and interspecies transmissions.

In this study, the estimated *R*_0_ values were in the range 1.45–2.12 between animals in the CLM production system with an average value of 1.68. This value is higher than the *R*_0_ of 1.45 previously reported in Ethiopia in the CLM production system from sero-prevalence data [18]. It is also higher than the report from sheep to cattle (1.0) [17] and from sheep to sheep (1.1) [9] and lower than other previous reports in intraspecies transmission in cattle of 2.52 [8] and ∞ (1.3−∞) [39]. As reported in the previous studies, the transmission from cattle to cattle is higher than sheep to cattle and cattle are more infectious than sheep [8, 17]. The cattle population in the CLM production system in the current study is higher (82%) than sheep and goat population (18%) and this may increase the transmission rate between these mixed populations than the sheep to sheep and sheep to cattle transmission reported in the previous studies. The *R*_0_ in the CLM production system in the current study is lower than the *R*_0_ in the commercial dairy farms; this is due to the high rate of contact of cattle in the commercial dairy farms.

The *R*_0_ values within the commercial dairy farms in the current study were in the range of 1.26–2.52 with an average value of 1.98. These *R*_0_ values were lower than the *R*_0_ value of 4.4 quantified from both direct and indirect transmission and 3.7 from direct transmission reported from experimental studies in calves in the Netherlands [11] and *R*_0_ value of ∞ (1.3−∞) in unvaccinated cattle in the Netherlands [39]; while it was close to the *R*_0_ value of 2.52 reported in cattle in the Netherlands [8]. The difference might be explained by the study type, different study population, the environmental difference and the production system set up.

In the current study, the average transmission rates were medium, 0.26 and 0.33 per day between animals in CLM production system and the commercial dairy farms, respectively. The estimated between-animals *R*_0_ was greater than the threshold level and moderate both in the CLM production system and in the commercial dairy farms. The moderate *R*_0_s estimated here indicate the vaccination coverage required to stop the disease transmission is not very high. In the CLM production system, the *R*_0_ was quantified for a mixed population of cattle, sheep and goat, but does not estimate the contribution of each species to the total transmission. Therefore, future research should focus on the estimation of the contribution of each species of animals for the transmission of FMD and on the implementation and evaluation of effective control measures that bring *R*_0_ below the threshold level.
